# Editorial: Antimicrobial Resistance and Virulence Common Mechanisms

**DOI:** 10.3389/fmicb.2017.00310

**Published:** 2017-03-03

**Authors:** Etienne Giraud, Ivan Rychlik, Axel Cloeckaert

**Affiliations:** ^1^ISP, Institut National de la Recherche Agronomique, Université François Rabelais de Tours, UMR 1282Nouzilly, France; ^2^TOXALIM (Research Centre in Food Toxicology), ENVT, INP-Purpan, Institut National de la Recherche Agronomique, UPS, Université de ToulouseToulouse, France; ^3^Department of Immunology, Veterinary Research InstituteBrno, Czechia

**Keywords:** antimicrobial resistance mechanisms, virulence, co-evolution, biofilms, fitness costs

Multiple relationships exist between antimicrobial resistance and bacterial virulence, and the spread of clones combining multiple antibiotic resistance and a high virulence level is an increasing problem. These relationships have been previously reviewed, notably by Beceiro et al. (2013), who described how mutation-driven or horizontally acquired resistance mechanisms can also have effects on virulence. It was also reported that mobile genetic elements often carry both resistance determinants and virulence-modulating genes, which favors the co-selection of both traits. In the present volume, we present a collection of articles which document additional aspects of the interactions between antimicrobial resistance and virulence in bacteria, and describe their potential therapeutic consequences.

In an excellent review article, Alcade-Rico et al. established that multidrug efflux pumps are “at the cross-road between resistance and virulence of bacterial pathogens.” They describe how multidrug efflux pumps are involved, besides antibiotic resistance, in many physiological and virulence-related processes controlled by complex regulatory networks. A special focus is made on the role of these efflux pumps in bacterial cell-to-cell communication and in bacterial-host interactions.

Another example of link between resistance and virulence in *Staphylococci* is given in a mini review by Qin et al. The authors summarized how the *psm-mec* locus present in some methicillin-resistant *Staphylococcus aureus* (MRSA) and coagulase-negative *Staphylococci* influences methicillin resistance and virulence-related phenotypes, including the ability to form biofilms.

In another paper also dealing with biofilms, Wang et al. used an up-to-date iTRAQ protein labeling technology to identify proteins that are differencially expressed in *Streptococcus suis* treated with sub-MIC concentrations of tylosin. These concentrations were indeed capable of inhibition of biofilm formation by this zoonotic pathogen, which is of interest, since biofilms are known to display an increased tolerance to antibiotics.

This antibiotic tolerance of bacteria present in biofilms is thought to be at least partially due to the presence of higher numbers of persisters than in planktonic bacterial populations. Persisters are phenotypic variants of bacteria which are able to survive antibiotic treatments in a dormancy state and to resume growth when the (antibiotic) stress is withdrawn. Like antibiotic-resistant bacteria, antibiotic-tolerant persisters are also often responsible for failures of antibiotic therapies. However, the mechanisms underlying their production are still not well-understood. An article by Xu et al. lifts a corner of the veil on this subject in *Staphylococcus aureus* by suggesting that heme biosynthesis, in addition to being necessary to a full expression of virulence, is also important in the formation of persisters in *S. aureus*.

Another study by Chen et al. addressed the problem of antibiotic tolerance in extraintestinal pathogenic *Escherichia coli* (ExPEC). Authors studied the role of polyphosphate kinase (PPK), an enzyme already known for its implication in motility, quorum sensing or virulence, in antibiotic tolerance. Their results indicate that PPK is important for the antibiotic stress response and may therefore be a valuable antibacterial target, especially since it is absent in mammals.

Small Colony Variants (SCV) represent another state in which bacteria are more tolerant to antibiotics. The clinical importance of SCV has been well-documented in cases of chronic infections by *S. aureus* and *Pseudomonas aeruginosa* but their existence was only recently reported in *Listeria monocytogenes*, a food pathogen producing recurrent infections despite being rarely resistant to antibiotics. In an original research article, Curtis et al. described a heme mutant displaying a SCV phenotype mutant with an increased tolerance toward most of the relevant antibiotics. They suggest that the SCV phenotype of *L. monocytogenes* should be screened in clinical laboratories, as it might be associated to complicated antibiotic treatments.

Genetic changes leading to antimicrobial resistance are expected to result in higher fitness costs in the absence of antibiotic selection pressure. On the other hand, increased resistance is sometimes associated with increased virulence or to a better adaptation to stress conditions. This topic is developed in a study by Schaufler et al. who reported that the carriage of extended spectrum beta-lactamase (ESBL)-plasmids by strains of pandemic *E. coli* lineages did not lead to a fitness cost. Instead, these plasmids could enhance virulence or adaptation to specific habitats by influencing the expression of chromosomal genes.

In another article dealing with bacterial fitness, Agnello et al. presented results that possibly explain why highly virulent *P. aeruginosa* strains, which often possess the *exoU* toxin gene, are more frequently resistant to fluoroquinolones than the generally less virulent strains harboring the *exoS* toxin gene. Their results support the hypothesis that fitness cost imposed by fluoroquinolone resistance may be lower and “more easily” compensable by *exoU* strains than by *exoS* strains.

In a whole genome sequence-based study, Hao et al. analyze a multidrug resistant and virulent *Campylobacter jejuni* strain isolated from a broiler chicken. By comparison to reference strains, they revealed that the phenotype of this isolate was associated with large differences in the genome structure and content. This study provides an example of how a stepwise accumulation of mutations in antibiotic target genes, acquisition of exogenous resistance genes and virulence associated genes can result in the emergence of isolates that are both multidrug-resistant and virulent.

Since quorum sensing is a major player in the control of virulence factors in *P. aeruginosa*, therapies based on quorum sensing interference (QSI) have been envisaged as possible alternatives to conventional antibiotic therapies against this pathogen. In a thoughtful perspective article, Garcia-Contreras, however, compiles many recent experimental evidence which actually indicate that early optimistic expectations about QSI should be put in perspective. Indeed, he estimates that a far better understanding of *P. aeruginosa* virulence and behavior during infection is needed before efficient QSI-based clinical applications can be designed.

In addition to some immunomodulating activities, azithromycin (AZM) has various inhibiting effects on *P. aeruginosa* such as bacterial killing or the repression of multiple virulence factors. This antibiotic is therefore currently used in the treatment of cystic fibrosis patients, whose lungs are commonly colonized by this pathogen. However, *P. aeruginosa* responds to AZM by yet unknown mechanisms and is able to counteract its killing and virulence-inhibitory effects. An interesting work by Tan et al. revealed that PA3297, a *P. aeruginosa* gene encoding a DEAH-box helicase, is involved in this response. Indeed, deficiency of this gene renders *P. aeruginosa* more susceptible to the killing and to virulence suppression by AZM. Authors therefore suggested that targeting the regulatory pathway of PA3297 or its function might increase the beneficial effect of AZM in chronic *P. aeruginosa* infections such as those occuring in cystic fibrosis patients.

At last, in a pharmacological perspective, Obtreska-Machaj et al. report the development of hydantoin derivatives that have increased activity as inhibitors of the AcrAB-TolC efflux pump of *Enterobacter aerogenes*. Close homologs of this multidrug pump are also present in other pathogenic enterobacteria and involved in their virulence. The authors defined important pharmacophoric groups whose modulation may allow to develop still more potent inhibitors capable of restoring antibiotic activity in AcrAB producing bacteria.

It is likely that both resistant and virulent high risk clones will continue to emerge and spread in the next years. Several factors supports this hypothesis, among which the facts that highly pathogenic bacterial strains are more likely to be subjected to the selective pressure of antibiotics treatments, that virulence and resistance determinants are sometimes genetically associated and that fitness and virulence impairments that often accompany acquisition of resistance can be compensated in the long term by suppressor mutations (Beceiro et al., 2013; Mathers et al., 2015). In spite of these facts, virulence and antimicrobial resistance are studied usually separately rather than jointly. But now, as exemplified in this research topic, time has come for more integrative studies linking both traits and their co-evolution, so that we may be better armed against future bacterial threats.

## Author contributions

All authors have substantially contributed to this work and approved it for publication.

### Conflict of interest statement

The authors declare that the research was conducted in the absence of any commercial or financial relationships that could be construed as a potential conflict of interest.
